# From Chest Wall Resection to Medical Management: The Continued Saga of Parapneumonic Effusion Management and Future Directions

**DOI:** 10.7759/cureus.21017

**Published:** 2022-01-07

**Authors:** Ratnam K Santoshi, Prarthna Chandar, SushilKumar S Gupta, Yizhak Kupfer, Ory Wiesel

**Affiliations:** 1 Critical Care Medicine, Maimonides Medical Center, Brooklyn, USA; 2 Pulmonary Critical Care, Thomas Jefferson University Hospital, Philadelphia, USA; 3 Pulmonary and Critical Care Medicine, Cayuga Medical Center, Ithaca, USA; 4 Critical Care, Maimonides Medical Center, Brooklyn, USA; 5 Thoracic Surgery, Maimonides Medical Center, Brooklyn, USA

**Keywords:** pleural effusion, tuberculosis, thoracentesis, pleural space infections, thoracostomy, empyema, vats

## Abstract

Pleural space infections have been described since the time of Hippocrates and to this day remains a significant pathology. Every year in the USA approximately there are one million hospital admissions for pneumonia with 20%-40% associated with some form of pleural space infections leading to pleural effusions with increased morbidity and mortality. Often, management of these effusions mandate combination of medical treatment and surgical drainage with debridement and decortication. There has been a lot of ongoing research regarding the safety and efficacy of intrapleural fibrinolytics in the management of complicated pleural effusions and empyema. Till this day, areas of debate and controversies exist among clinicians treating pleural space infection. Empyema is historically considered a surgical disease. There have been societies and guidelines for the management of infected parapneumonic effusions with antibiotics and chest tube drainage as an initial empiric treatment modality. With the advances in the use of Intrapleural fibrinolytics and minimally invasive procedures such as video-assisted thoracoscopic surgery (VATS), empyema a surgical disease is now more favoring medical management. Surgical option, such as open thoracotomy, is reserved for patients who failed conservative management and chronic empyema. The aim of this comprehensive review is to shed light on the evolution of various management strategies from the era of Hippocrates to current day practice and how there continues to be a paradigm shift in treating empyema as a surgical condition to a medical disease.

## Introduction and background

Introduction of parapneumonic effusion

The pleural space is a potential space between the two pleurae: visceral and parietal pleura of the lungs. Under normal circumstances, there is no soft tissue or free air in this space. However, a small amount of clear pleural fluid (approx. 10-20mL) with a low protein concentration (<1.5g/dL) may be present. Parapneumonic effusion is an accumulation of fluid in the pleural space secondary to pulmonary infection and occurs as a common complication of pneumonia in up to 57% of cases [1]. Parapneumonic pleural effusions are classified into three types based on the characteristics of the pleural fluid such as uncomplicated parapneumonic effusion, complicated parapneumonic effusion, and empyema thoracis. Uncomplicated parapneumonic effusion accounts for most pleural effusions which are characterized by clear, sterile exudates that frequently resolve with antibiotics alone and do not require drainage. Complicated parapneumonic effusions contain fibrin deposition, fluid is infected but not purulent, does not isolate any microorganisms, and contains increased neutrophils, decreased glucose levels, elevated LDH, and pH < 7.20. These effusions do not resolve without fluid drainage. Empyema is persistent pleural infection with accumulation of pus in the pleural space [1]. Pleural space infections have been described since the time of Hippocrates and to this day remains a significant pathology [2]. Pleural space infections can be secondary to infectious processes in the lung (parapneumonic effusion) or the spread of infection from penetrating chest trauma, instrumentation, abdominal infection, or surgery, and from injury to mediastinal organs such as esophageal injury. While morbidity and mortality have improved, the incidence of pleural space infections is on the rise [3]. Every year in the USA approximately there are one million hospital admissions for pneumonia and 20%-40% of these are associated with some form of pleural space infections [3]. Morbidity and mortality associated with parapneumonic effusions are high, with more mortality seen in the elderly and debilitated population. Pneumonias associated with parapneumonic effusions have significantly higher mortality than patients admitted with pneumonia without an effusion ranging between 10% and 20% [3]. Oftentimes, management of these effusions mandates a combination of medical treatment and surgical drainage, decortication, and debridement. Although heavily studied, there is no consensus on the proper management of para-pneumonic effusion. The aim of this comprehensive manuscript is to review the current literature with an emphasis on the current consensus and areas of continued research in the management of these complicated effusions.

Historical perspective of parapneumonic effusion

The oldest known reference dates to 2,400 years ago and was first attributed to Hippocrates [2]. In the Hippocratic texts- “empyema’s” could occur in any part of the body and were not distinguished from abscesses, although the “empyema’s” located in the thorax were described most often [2]. In 229 BC, Hippocrates described a patient with empyema with an emphasis on physical examination and clinical findings. The first drainage procedure for empyema was also performed by Hippocrates and it was done by partial rib resection, drainage, and daily packing. It was not until later in the 18th century that additional description about the pathogenesis and treatment of empyema was described in the literature. In 1879, Estlander described thoracoplasty and Fowler advanced the surgical approach and described the decortication as an integral part of empyema treatment [4]. Descriptions of thoracic wounds requiring drainage were described in the context of battlefield wounds and tuberculosis. Graham and Bell, of the United States Army made a major advancement in the treatment of empyema. They recommended closed tube drainage to manage early empyema which decreased the mortality rate from 30% to 4.3% [5]. In 1935, Eloesser described an open thoracotomy technique that would permit skin and soft tissue to behave as a valve and allow lung expansion; later in 1963, Clagett and Geraci introduced open window drainage for 6-8 weeks with antibiotic solution and packing followed by cavity obliteration and closure [6]. Since then various advancements and refinement of the drainage techniques including fibrinolytic and video-assisted thoracoscopic surgery (VATS) have been developed with a better understanding of pulmonary physiology. In countries where mycobacterium tuberculosis (TB) is endemic, TB remains the main etiology for the development of non-traumatic empyema [6]. Management of tuberculous empyema has evolved from surgical drainage in earlier years to non-surgical management with the discovery of anti-tuberculous chemotherapy. Compared to patients with nontuberculous empyema, Tuberculous empyema patients have a protracted duration of illness, a significant incidence of bronchopleural fistula necessitating complicated drainage including surgical drainage, and a relatively poor outcome [6].

Pathophysiology of parapneumonic effusion

Pleural space infections are most commonly secondary to infectious processes in the lung. Other etiologies include post-surgical, instrumentation, post-traumatic, and extension of intra-abdominal infections. 52% are related to post pneumonic, 24% postresection, 14% as complications of minor surgical procedures, 5% posttraumatic, and 5% as a result of other miscellaneous causes [7]. Post resection empyema is attributed to 0.01% of sub-lobar resections, 2% of lobectomies, and up to 5% of pneumonectomies [7].

Stages of parapneumonic effusion

The three stages in the natural evolution of parapneumonic effusion include exudative, fibrinopurulent, and the organizing phase [8]. The exudative phase is characterized by rapid accumulation of pulmonary interstitial fluid in the pleural space primarily due to the inflammatory process initiated by the pneumonic process as well as an increased capillary permeability in the pleural space. The main inflammatory mediator implicated at this stage is the vascular endothelial growth factor (VEGF). VEGF receptors are found on the pleura’s mesothelial cells, and the levels of VEGF are higher in exudative effusion than in transudative effusion [9]. At this phase the pleural fluid is characterized by negative bacterial studies, pH > 7.20, glucose > 60mg/dL (3.3mmol/L), and a lactic acid dehydrogenase (LDH) level of fewer than three times the upper normal limit of serum. This state is also referred to as a non-complicated parapneumonic effusion [9]. In the fibrinopurulent phase, the pleural fluid is purulent with increased viscosity, rich in cellular debris, fibrin deposition, polymorphonuclear cells, and inflammatory cytokines (such as IL-1 and TNF-α). IL-1 induces mesothelial cells to release transforming growth factor (TGF-β) which is one of the most potent fibrinogenic agents. When the pleura is inflamed, the amount of fibrin that is laid down is the result of the homeostasis between fibrinogenesis and fibrinolysis. Fibrogenesis occurs when the factors that favor fibrogenesis such as TNF-α, TGF-β, and plasminogen activation inhibitor-1 (PAI-1) are dominant [9]. The pleural fluid at this stage is characterized by microbial infection, fibrin deposition, and the effusion color may now progress from clear yellow to purulent. Biochemical analysis of the pleural fluid reflects pH < 7.20, glucose < 60mg/dL (3.3mmol/L), LDH level of more than three times the upper normal limit of serum, or a positive microbial culture. The fluid is now referred to as complicated parapneumonic effusions or empyema [9]. Complicated parapneumonic effusions often develop fibrinous adhesions resulting in loculations, impeding the drainage of the simple pleural fluid. The fibrinopurulent stage is followed by the organizing phase where the solid pleural peel replaces the soft fibrin overlying the visceral pleura, which impedes lung re-expansion. Patients may have restrictive lung patterns on the pulmonary function test, and creating a persistent pleural space with “trapped” lung [9].

Microbiology of parapneumonic effusion

The knowledge of the most common causative organisms responsible for pleural space infections is important to clinicians to aid in appropriate empiric antibiotic treatment. An extensive systemic review done by Hassan et al. showed that the mean diagnostic yield of pleural fluid cultures is only 56% and hence antimicrobial treatment is mostly empiric [10]. The microorganisms responsible for causing pneumonias and pleural space infections are varied and this is believed to be due to the hypoxic nature of the pleural space which favors the growth of anaerobic bacteria over other organisms. Hence empiric coverage for anaerobes is recommended for pleural space infections and empiric coverage for atypical organisms such as Mycoplasma and Legionella with macrolides are not recommended for pleural space infections unlike community-acquired pneumonias [3]. The two most common determinants to predict the causative organism for pleural space infection are geographic distribution and community-acquired vs nosocomial [10]. Staphylococcus aureus is more prevalent worldwide, Pneumococci in tropics and Viridans streptococci in temperate zones [10]. Gram-positive aerobic organisms accounted for 50.4% which includes Staph. aureus, Strep. milleri (more recently termed “Streptococcus anginosus and was included in viridans streptococci group), Pseudomonas, Enterobacteriaceae group, Strep. Pneumoniae, Klebsiella, Acinetobacter species, and coagulase-negative staphylococci; Gram-negative aerobic organisms accounted for 37.5%, and anaerobes were 12.1% [10]. In community-acquired pleural infections, the most commonly isolated organisms are Viridans streptococci followed by Pneumococci, Staph. aureus, Enterobacteriaceae, Klebsiella, Pseudomonas, Beta hemolytic streptococci, Acinetobacter. In nosocomial settings, the most common organisms isolated are Staph. aureus followed by Enterobacteriaceae, Viridans streptococci, Pseudomonas, Klebsiella, Enterococci, Acinetobacter, Pneumococci. Methicillin-resistant Staph. aureus (MRSA) is common in nosocomial settings (67% vs 42% in community-acquired vs nosocomial) [10]. Infections from Strep. milleri groups have become the predominant organism cultured from adults with empyemas, especially in patients with underlying malignancy or diabetes mellitus [11]. The most common organism for pleural infection in a pediatric age group is Strep. pneumoniae. Staph. aureus infections are often seen in elderly hospitalized patients with multiple comorbidities. It has the propensity to cause cavitary lung lesions, lung abscesses, accounting for 10%-24% of adult empyema and up to 50% in a pediatric population. Clinicians should also be aware of recently emerging incidences of community-acquired MRSA empyema. Certain high-risk patients such as diabetes mellitus, malignancy, and liver cirrhosis are more prone to Gram-negative empyema [11]. The risk factor for anaerobic microorganisms is poor dentition which due to aspiration or via hematogenous spread can cause parapneumonic effusion and usually coinfect with aerobic organisms [12]. An exception to this is Pneumococci which does not co-infect with other microorganisms. The anaerobic bacteria are found in 70% of pleural fluid samples tested. Due to this high prevalence of anaerobes, it is recommended to have empiric anaerobic coverage for pleural space infections. Fungi are uncommon causative organisms for pleural infection accounting for up to 3% and candida is commonly isolated in immunosuppressed patients (Table 1) [10].

**Table 1:** Etiology of empyema and most commonly associated microorganisms

Geographic distribution and common microorganisms	
Staph. aureus	prevalent worldwide
Pneumococci	tropics
Viridans streptococci	temperate zones
Community vs Nosocomial	
Community acquired	Viridans streptococci followed by Pneumococci, Staph. aureus, Enterobacteriaceae, Klebsiella, pseudomonas, beta hemolytic streptococci, Acinetobacter
Nosocomial	Staph. aureus followed by Enterobacteriaceae, Viridans streptococci, Pseudomonas, Klebsiella, Enterococci, Acinetobacter, Pneumococci
Other Categories	
Staph. aureus	elderly hospitalized patients with multiple comorbidities
Gram-negative	diabetes mellitus, malignancy, and liver cirrhosis
Anaerobic microorganisms	poor dentition
Candida	immunosuppressed patients

Clinical presentation of parapneumonic effusion

Clinical presentation depends on the patient’s timing of presentation and immune competency. Patients with pneumonia and uncomplicated effusions present earlier while patients with empyema typically present later when the bacteria from untreated pneumonia had sufficient time to colonize the pleural space. Some common symptoms include cough, fever, pleuritic chest pain, dyspnea, fatigue, and sputum production. Compared to patients with pneumonia alone, patients with empyema typically report a longer course of symptoms and have more likelihood of hospital admission and significant morbidity and mortality. Physical examination typical of effusion is dullness to percussion over the fluid, crepitus, and euphony at the upper level of the fluid [13]. In the prospective study by Chalmers et al. they identified six risk factors that were associated with patients admitted with community-acquired pneumonia who subsequently developed a complicated parapneumonic effusion or empyema. These factors include albumin < 30 g/L, sodium < 130 mmol/L, platelet count > 400 x 10^9^, C-reactive protein >100 mg/L, and a history of alcohol abuse or intravenous drug use [13]. Other predispositions include immunosuppression (e.g., HIV, diabetes mellitus, malnutrition), gastrointestinal reflux, poor dental hygiene, bronchial aspiration, and chronic lung disease. The predisposing risk factors for empyema are listed in Table 2.

**Table 2:** Predisposing risk factors for empyema

Primary lung disorders	Instrumentation
Pneumonia Septic emboli Bronchiectasis (Klebsiella predominance) Cystic lung disorders Cavitating lung lesions Cavitating lung cancer Broncho-pleural fistulas	Superinfection of previous sterile effusion Thoracic surgery Cardiac surgery
Gastrointestinal related	Infection
Poor dentition Aspiration tendency Inability to swallow/protect airway (poor gag reflex) Hiatal hernia, GERD Esophageal dysmotility disorder (Achalasia) Esophageal strictures	Primary lung infection Spread from mediastinal infection Spread from abdominal infection
Trauma	Immunosuppression
Penetrating Chest wall injury Hemothorax Esophageal perforation	Mycobacterium infections
	Alcohol abuse

Imaging modalities for parapneumonic effusion

**Figure 1:**
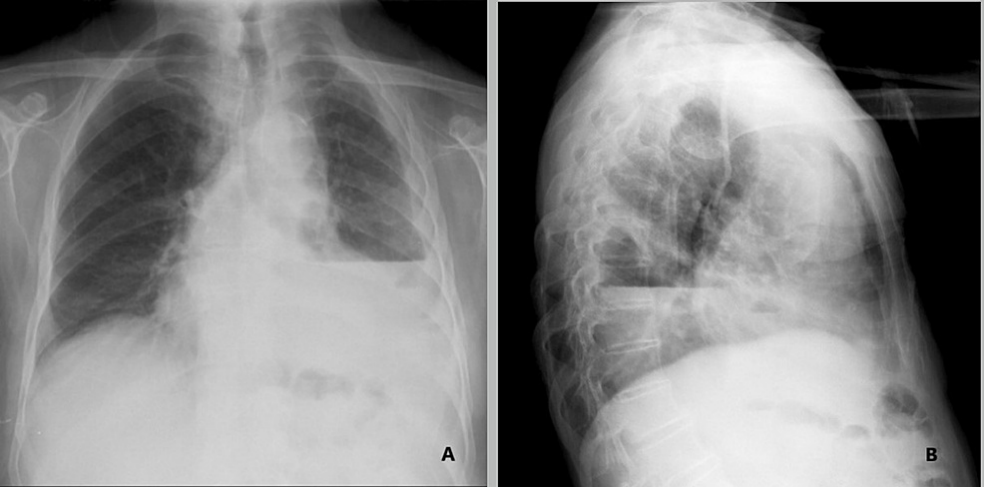
Chest x-ray showing pleural effusion with air-fluid level. (A) Anteroposterior view of the air-fluid level extending all the way to the chest wall. (B) Lateral view of the air-fluid level. Open access. Creative commons license. Case courtesy of Assoc Prof Frank Gaillard, Radiopaedia.org, rID: 15390

**Figure 2:**
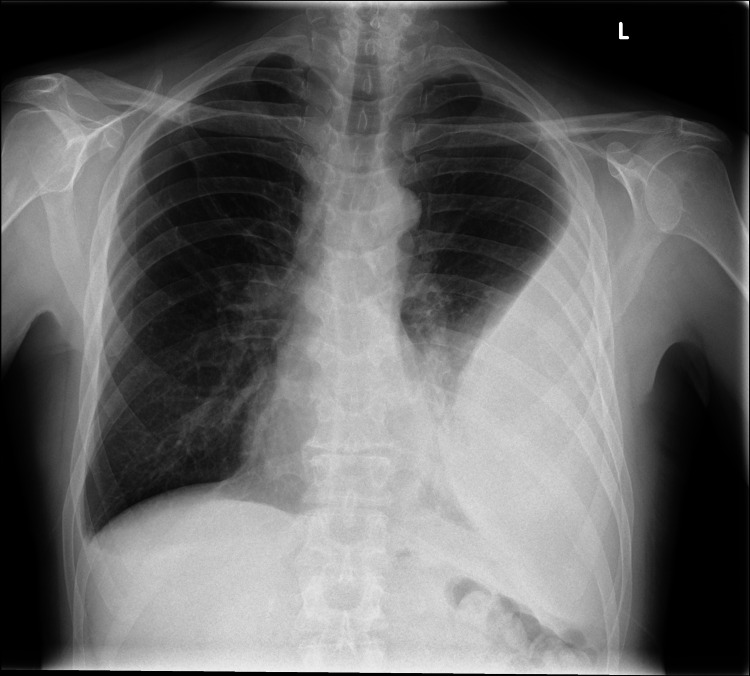
Left-sided biconvex pleural collection forming an obtuse angle with the chest wall. Open access. Creative commons license. Case courtesy of Dr Ian Bickle, Radiopaedia.org, rID: 74921

**Figure 3:**
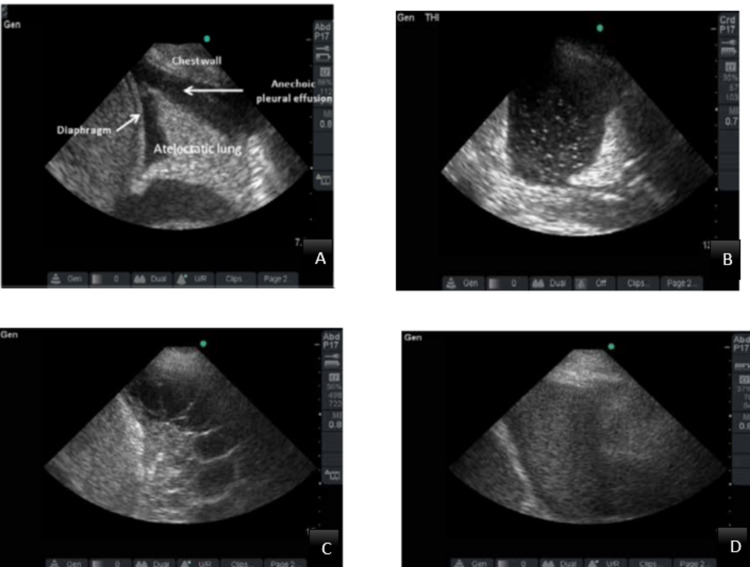
Ultrasound of lungs showing pleural effusion. (A) Pleural effusion is present as an anechoic space with anatomic boundaries made up of chest wall, diaphragm, and atelectatic lung. (B) Complex non-septated pleural effusion with swirling echogenic debris (Plankton Sign). (C) Complex septated pleural effusion. (D) Homogenously echogenic pleural effusion. Image courtesy by Rahul Khosla, Pulmonary & Critical Care Medicine, Veterans Affairs Medical Center, Washington, DC, USA, Lung Sonography chapter 6. Open access. Creative commons license.

**Figure 4:**
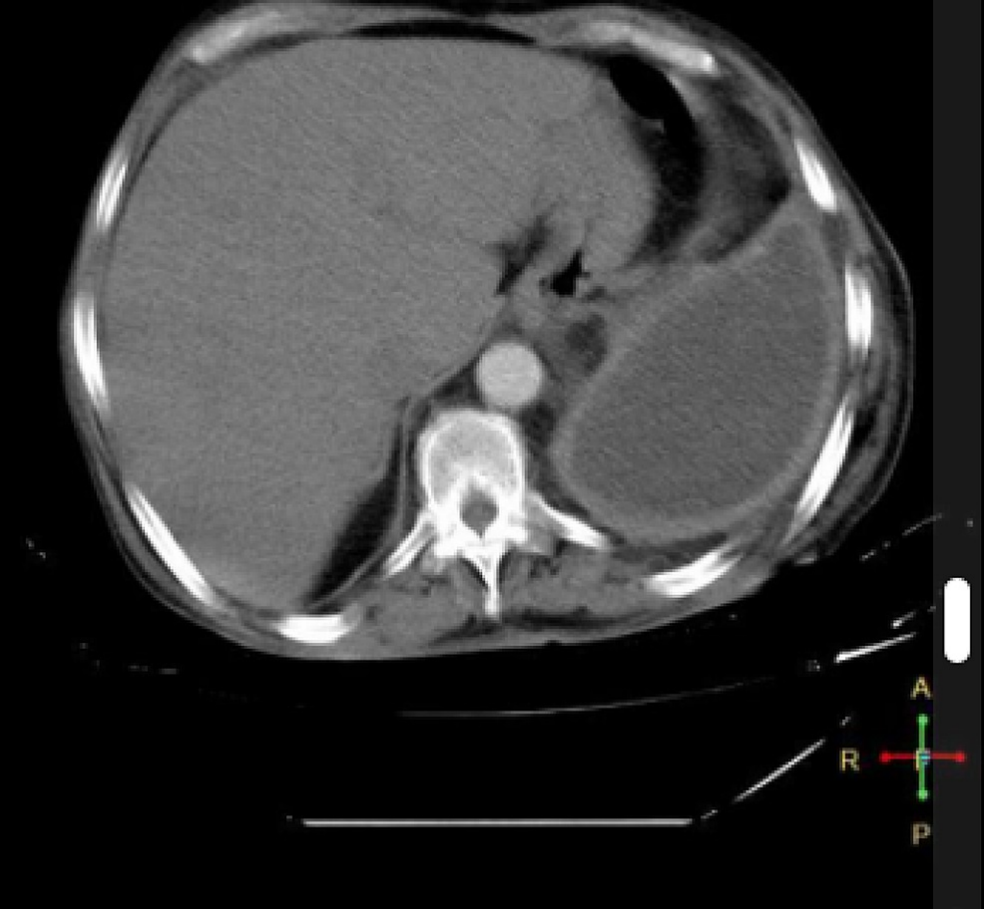
Split pleura sign on CT scan with thick pleural lining. Image courtesy of radiopedia. Creative Commons Attribution-Share Alike 3.0 Unported license. Case courtesy of Dr Ahmed Abdrabou, Radiopaedia.org, rID: 24442

Radiographic and ultrasound imaging play a vital role in the evaluation and management of parapneumonic effusions. Pleural effusions appear in the dependent positions of the chest and change with the patient’s position. Loculated effusions tend to occur secondary to pleural adhesions and scars. A small effusion causes blunting of costophrenic angles. Empyema has a lenticular shape and forms an obtuse angle with the chest wall, therefore on a chest radiograph, an empyema appears as a wide air-fluid level on posteroanterior view and has narrow anteroposterior width on lateral projections (Figures 1A, 1B, 2) [14]. Lateral decubitus films can also provide information regarding a free-flowing or loculated empyema. Ultrasound offers point-of-care imaging and can be used to delineate loculated effusion, quantify the volume, be more sensitive to identify septations, and guide thoracentesis. “Plankton sign” on ultrasonography is characterized by echogenic debris swirling within the pleural fluid and is seen most commonly in empyema, hemothorax, chylothorax, or exudative fluid from malignancy or other causes; however, the quality of the imaging depends on the operator (Figures 3A-3D). Computed tomography (CT) remains the gold standard imaging to diagnose empyema. In empyema, there is fibrin deposition on the parietal and visceral surfaces of the pleura resulting in the ingrowth of the blood vessels. In a contrast-enhanced CT, the empyema fluid separates the two pleural surfaces, forming a “split pleura sign” (Figure 4). It is the most sensitive and specific sign on CT and is helpful in distinguishing an empyema from a peripheral lung abscess, which has an irregular inner surface in contrast to a smooth surface in empyema [14,15].

## Review

Management of parapneumonic effusions

Treatment of parapneumonic effusions should be tailored based on the complexity of effusion, the stage of effusion, the patient’s clinical state, and comorbidities. Evacuation of the infected pleural fluid by a chest tube, in conjunction with antibiotic therapy, has since been the cornerstone of management. However, a complicated pleural fluid can be difficult to drain due to the presence of fibrous septations and the high fluid viscosity. Multiple societies such as The American College of Chest Physicians (ACCP) 2000, British Thoracic Society (BTS) 2010, The American Association for Thoracic Surgery (AATS) 2017, published guidelines for the management of parapneumonic effusions [3,16,17]. Empyema is historically considered a surgical disease and treatment of empyema and complicated parapneumonic effusions with open thoracic drainage is a gold standard for many years and is traced back to the Hippocrates era [2]. The introduction of intrapleural fibrinolytics (IPF) and minimally invasive strategies such as VATS have been extensively studied. There is an area of ongoing research with regards to the timing, selection of patients, dosage, and safety profile for the routine use of these modalities in the management of complicated parapneumonic effusions.

Antibiotic treatment

A diagnostic thoracentesis is usually needed to evaluate the nature of the effusion; transudative versus exudative, obtaining gram stain and culture results. Empiric antibiotic therapy should be selected based on the patient’s clinical presentation, local antimicrobial resistance pattern, antibiotic stewardship of the institution, and pharmacological characteristics. A parenteral second- or third-generation cephalosporin such as ceftriaxone with metronidazole for anaerobic coverage or parenteral aminopenicillin with b-lactamase inhibitor (e.g., ampicillin/sulbactam) is empiric for community-acquired pleural empyema [3,17]. Antibiotics against MRSA and anti-Pseudomonal coverage are considered for hospital-acquired or post-procedural empyema. Antimicrobial agents covering anaerobic infection should be used empirically except those with culture-proven pneumococcal infection. Clindamycin is an alternative to metronidazole for anaerobic coverage for those who are sensitive or cannot tolerate it. Macrolide antibiotics are not indicated unless there is objective evidence for or a high clinical index of suspicion of ‘atypical’ pathogens. Most antibiotics have good pleural penetration except for aminoglycosides which may be inactivated at low pleural fluid pH [3,17]. Carbapenems can be added if there is a history or suspicion of extended-spectrum b-lactamase producing organisms (Table 3) [3].

**Table 3:** Etiology of empyema and choice of empiric antimicrobial

Etiology	Antimicrobials
Community-acquired empyema	Parenteral second- or third-generation cephalosporin such as ceftriaxone with metronidazole for anaerobic coverage or parenteral aminopenicillin with b-lactamase inhibitor (eg, ampicillin/sulbactam)
Hospital-acquired or post-procedural empyema	Antibiotics against MRSA and anti-Pseudomonal coverage
History or suspicion of extended-spectrum b-lactamase producing organisms	Add Carbapenems

Duration of antibiotic therapy depends on various factors including response to therapy, the sensitivity of the organism, extent of parenchymal and pleural disease, adequacy of drainage, and host immune status [3,17]. A minimum of two weeks of antibiotic treatment post drainage and defervescence is recommended by The American Association for Thoracic Surgery Consensus guidelines. However, the total duration of antibiotic therapy has not been determined through comparative trials. Various factors that help determine the total duration are culture and sensitivities of the infecting organism, drainage efficacy, and clinical response to therapy. Intravenous antibiotics should be changed to oral therapy once there is clinical and objective evidence of response to the antibiotic regimen and the patient can tolerate oral intake. Chronic empyema should be managed closely by infectious disease specialists. There is no role for intrapleural administration of antibiotics in clearing bacterial infection or improved outcomes when compared to systemic antibiotics [3].

Thoracentesis

Thoracentesis is the drainage of the pleural fluid. Most parapneumonic effusions with less than 10mm fluid on CXR usually resolve with antibiotics. Diagnostic thoracentesis should be performed for pleural effusions larger than 10mm on a later decubitus radiograph or if there are signs of ongoing sepsis, associated with underlying pneumonia, recent surgery, or trauma to the chest. In cases of congestive heart failure, diagnostic thoracentesis is only indicated if the patient is febrile or has pleuritic chest pain, unilateral effusion, or effusions of markedly disparate size or the effusion fails to respond to management of the heart failure [17]. Ultrasound-guided pleural fluid drainage for diagnostic or therapeutic indications has been found to increase yield and patient safety. A recent AUDIO study demonstrated that ultrasound-guided pleural biopsy increases the microbiological yield to 45% compared to 20% by pleural fluid and 10% by blood cultures. The high yield by pleural biopsy was explained by the localization of the bacteria to the parietal pleura that has better blood supply instead of the acidic, hypoxic, and nutritionally deficient pleural fluid [18].

Tube thoracostomy

Tube thoracostomy is the placement of a chest tube in the pleural cavity to drain the infected pleural fluid and is recommended for the management of all complicated parapneumonic effusion and empyema. The British thoracic society guidelines and The American Association for Thoracic Surgery consensus guidelines recommend chest tube placement for pleural fluid pH < 7.2, frank pus, or turbid pleural fluid, presence of organisms by gram stain. When pleural fluid PH is not available, glucose of <3.4mmol/L or LDH > 1,000IU/L are indicators for chest tube placement. Physicians should be cautious of noninfectious etiology such as rheumatoid pleural effusions with low glucose levels and certain factors that can falsely reduce pH such as lidocaine and increase pH such as proteus infections [17]. Chest tubes can be placed under ultrasound guidance at the bedside or with computed tomography guidance by interventional radiologists. Loculated effusion and drainage of large non-purulent pleural effusion for symptomatic relief can be considered for chest tube placement [17]. Studies have shown that small-bore chest tubes 10-14F are non-inferior to large-bore chest tubes in efficacy and with minimal pain, however, concern for tube clogging with viscous effusions remains high. The patency of the small-bore flexible chest tube can be facilitated by flushing the catheter with 20 -30ml saline every six hours via a three-way tap. Resolution of pleural fluid, clinical improvement, and decreased output from the chest tube are indications for removal of the chest tube [3,17,19].

Intrapleural fibrinolytics (IPF)

When parapneumonic effusions reach the stage of fibrinopurulent phase, the fluid is very difficult to drain by chest tubes and these patients might be candidates for agents that will help lysis of the fibrin adhesions and decrease the viscosity of the fluid to help drain the effusions better. The role of the intrapleural installation of streptokinase, a fibrinolytic agent and desoxyribose nuclease has been described in the literature in 1949 to facilitate pleural fluid drainage [20]. Since then, there has been extensive research in this area with smaller case studies. However, these small case series did not have the statistical power to accurately evaluate important clinical outcomes, including safety, and feasibility, which led to the design of Multicenter Intrapleural Sepsis Trial 1 (MIST1) trial in 2005 in the United Kingdom [21]. MIST1 was a double-blind randomized trial that included 430 patients with pleural effusion. Patients were randomly assigned to receive either intrapleural streptokinase (250,000 IU twice daily for three days) or a placebo. The primary endpoint was 90 days mortality and secondary outcomes included the need for surgery, radiological changes, and the hospital length of stay. The trial concluded that intrapleural streptokinase neither decreased mortality, nor did it decrease the need for surgical drainage, or the hospital length of stay. The failure of mono therapeutic agents is attributed to the fact that complicated parapneumonic effusions not only contain fibrinous adhesions but also are highly viscous. The high viscosity is thought to be due to the presence of the deoxyribose nucleoprotein released by the breakdown of the leukocytes. This information led to the design of another randomized trial known as MIST2 in 2011, which used a different direct-acting fibrinolytic agent; recombinant tissue plasminogen activator (tPA) to lysis the fibrin along with recombinant human DNAase to reduce viscosity. In MIST2 trial a total of 210 patients were randomized into four groups: tPA plus placebo (n=52), DNase plus placebo (n=51), both agents (n=52), or double placebos (n=55). Combined intrapleural tPA/DNase resulted in a significantly greater reduction in radiographic pleural opacity, lower need for surgical intervention, and decreased length of hospital stay compared with placebo and single agent. The regimen used in this study was 10mg tPA and 5mg DNase twice a day for three days [22]. Since MIST2 trial, at least 11 non-randomized studies with over 600 patients with pleural infections were treated with this combination regimen and reported 87% cure without the need for rescue surgical intervention and less than 4% non-fatal pleural bleeding complications [23]. However, the safety and efficacy of these agents have been questioned. There is continued concern about potential bleeding risks from tPA use. Additionally, tPA is an expensive drug that accounts for 75% of combined costs of 10mg tPA plus 5mg DNase regimen precluding its routine use [24]. The dose of tPA in the MIST2 trial was empirically chosen without conventional dose escalation studies and given the increasing use of the combination regimen with high success rates, in 2016 Popowich et al. conducted a pilot study for Alteplase Dose Assessment for Pleural infection Therapy (ADAPT) project with a reduced dose of 5mg tpa and 5mg of DNase twice a day which showed to be as safe and effective regimen as previous studies. This study showed that <5% patients required blood transfusions for gradual pleural bleeding without hemodynamic instability which was comparable to previous studies using 10mg tPA dosing. In this study 11% patients needed dose escalation to 10mg tPA; nonetheless, this provides evidence that 5mg tPA can be an initial dose with much safety and efficacy. The use of intrapleural therapy with 10mg tpa and 5mg DNAase twice a day for six doses as described in the MIST2 trial was further studied by McClune et al. in 2016 to consider the extended use of these agents as an alternative treatment modality in patients who are poor surgical candidates and/or refuse surgical treatments [25]. The study involved a retrospective chart review of 101 patients at two institutions who received intrapleural therapy with tpa and DNAase with standard dosing of up to 6 doses and 20% patients received extended therapy beyond six doses with a mean of 9.8 (range of 7-16). The study showed no significant differences in complications, outcomes, or need for surgical procedures in either group. More research involving large RCT is warranted to understand the efficacy of these agents for extended therapy among frail patients who cannot tolerate surgery.

Video-assisted thoracoscopic surgery (VATS)

**Figure 5:**
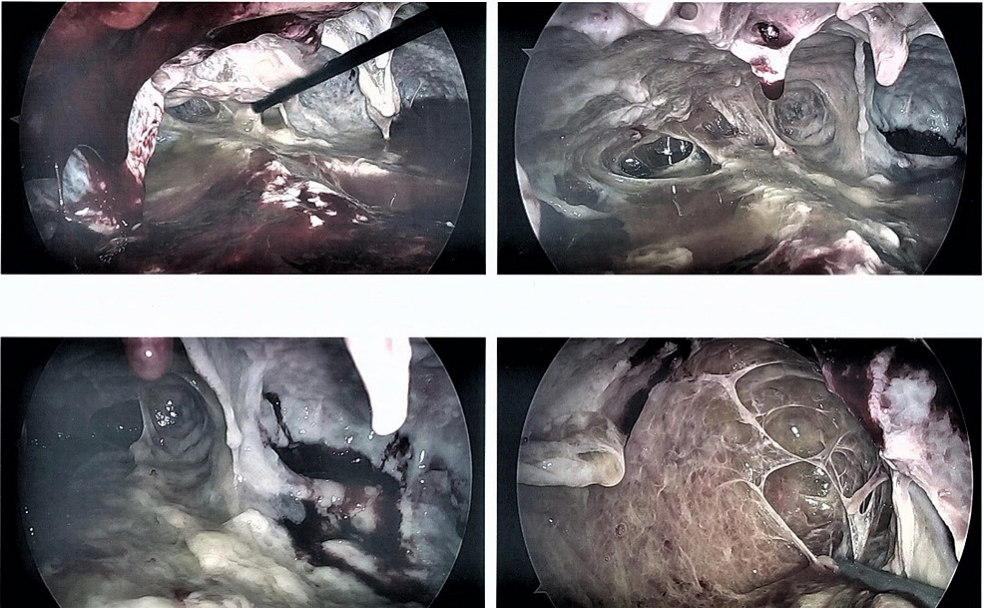
VATS for empyema. 62 yr M with empyema. Following the failure of drainage by chest tubes, the patient underwent VATS with drainage and decortication of the pleural surfaces: extensive pleural adhesions and pus in pleural space. Images Courtesy by Dr. Ory Wiesel VATS - Video-assisted thoracoscopic surgery

**Figure 6:**
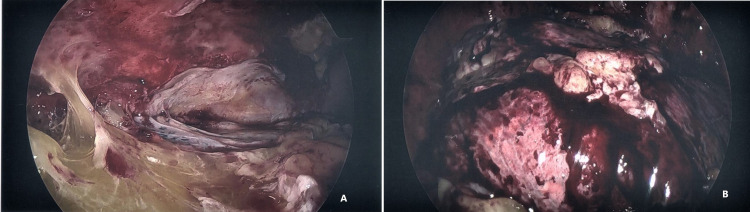
VATS for empyema. 41 yr M with empyema underwent VATS. (A) Found to have multiple loculated collections and underwent decortication for empyema. (B) Note the beginning of the expansion of the previously trapped lung following decortication. Images Courtesy by Dr. Ory Wiese VATS - Video-assisted thoracoscopic surgery

**Figure 7:**
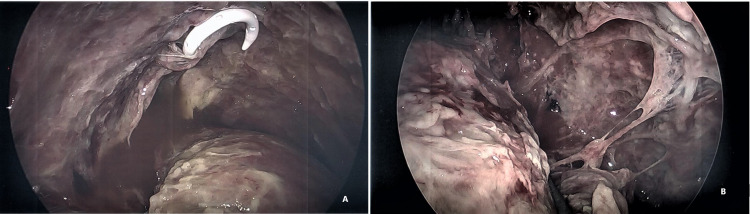
VATS for fibro purulent parapneumonic effusion. (A, B) 69 yr M with fibro purulent parapneumonic effusion of the left pleural space. Underwent VATS washout and decortication. Note the pigtail drain in the pleural space which was inserted earlier for conservative management. Images Courtesy by Dr. Ory Wiesel VATS - Video-assisted thoracoscopic surgery

When the third phase of empyema, the organizing phase develops, there is the formation of the thick fibrous pleural peel which encases the lung, preventing re-expansion of the lung and creating trapped lung physiology. Medical therapies are likely to fail in this phase. When patients do not improve with antibiotics and adequate drainage of pleural fluid with tube thoracostomy and have persistence or worsening of parapneumonic effusion; early consultation with thoracic surgeons to discuss all surgical options should be considered [17]. The two minimally invasive procedures currently available are medical thoracoscopy and VATS. VATS is considered to be a procedure involving minimal rib distraction and less damage to the intercostal neurovascular bundle [3]. The preferred method at recent times is VATS as it provides the operator with much larger access to the pleural space. VATS has been found to be an effective method in the management of fibrinopurulent and organizing stages of empyema [26]. VATS offer a minimally invasive approach through one to three side ports and necessitates fewer hospital days and morbidity than open surgical procedures (Figures 5-7A, 7B) [27]. The main goals of surgical therapy are Triple D’s - “drain, de-loculate and debride”; Drainage of the free effusion, Deloculate and drain all loculated effusions pockets and Debridement and Decortication of all pleural surfaces to allow proper lung expansion.

Open thoracostomy procedures

**Figure 8:**
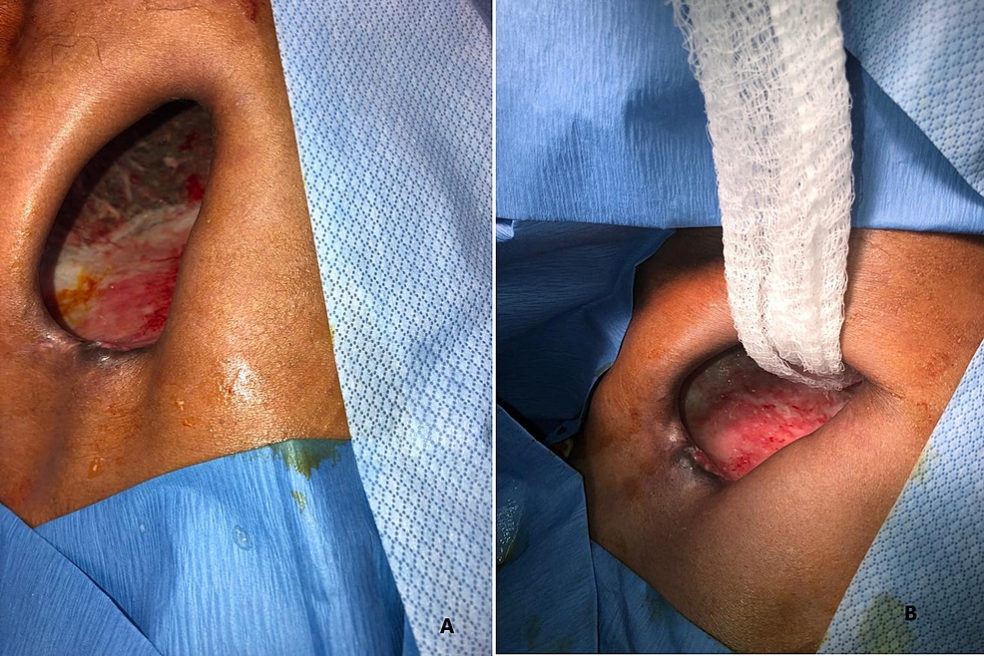
Clagett's procedure. (A, B)  57 yr, Immuno-compromised patient with h/o stage IIIa lung cancer s/p Chemoradiation and left VATS upper lobectomy, who subsequently developed trapped lung and chronic empyema, s/p left VATS, decortication, and Claggett’s procedure with multiple washouts for persistent bronchopleural fistula. Images Courtesy by Dr. Ory Wiesel

**Figure 9:**
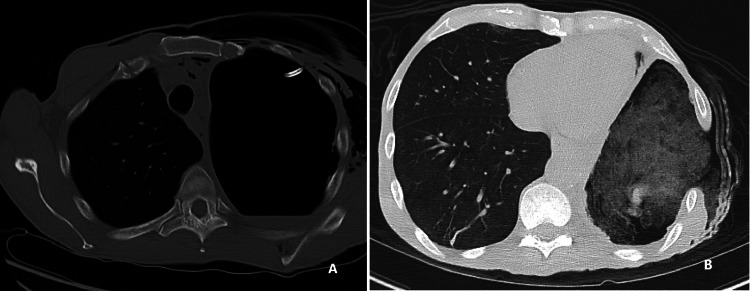
CT scan chest of the same patient above showing post left upper lobectomy. (A) Infected pleural space with multiple pleural adhesions and air-fluid level in the left lung base. Atelectasis in the remaining left lower lobe. Soft tissue emphysema of the left hemithorax. S/p chest tube for empyema drainage. (B) CT chest of the same patient five months later showing left upper lobectomy seen previously and interval creation of a left Claggett window. Packing material filling the left pleural space is associated with atelectasis of the left lower lobe. No collections identified in the left hemithorax. Images Courtesy by Dr. Ory Wiesel

Despite the benefit of minimally invasive surgery, open thoracotomy might be necessary for long-standing infection with fibrothorax. Other indications for open thoracostomy are listed in Table 4. Patients who failed conservative measures and who are not candidates for further decortication procedures due to poor cardiopulmonary conditions need open drainage procedures [3] Two procedures classically described are Eloesser flap (Figures 8A, 8B) [7] and Clagget window (Figures 9A, 9B) [8].

**Table 4:** Indications for open thoracostomy

Persistent and chronic empyema that failed the first-line management with surgical pleural space drainage and decortication procedures
Persistent broncho-pleural fistula (BPF)
Persistent tuberculous empyema
Postpneumonectomy empyema with or without BPF
Contraindications for early decortication procedures due to poor baseline cardiopulmonary/functional status and or poor surgical candidates

Eloesser flap was originally described in 1935 for tuberculous empyema. Clagget window described in 1963 for the treatment of Post pneumonectomy empyema. Both techniques are used today selectively for the treatment of chronic pleural space infection or as salvage for very frail patients or septic patients who cannot withstand debridement surgery. The concept of open thoracostomy is based upon durable drainage of the space which allows granulation tissue formation over time with repeated bedside dressing changes. Often, antibiotic solutions or bactericidal solutions can be used to improve local infection control. Three to 12 months of local wound care with daily packing is the standard of care. The wound usually heals with secondary intention over granulation tissue or is planned for a staged procedure with muscle flap (or omentum) with skin graft closure when appropriate infection control is ascertained. Optimum nutrition status is another key for wound healing.

Unique surgical circumstances

Tuberculous empyema: Tuberculosis, as old as humankind, is still a significant pathogen in many areas of the world. According to World Health Organization (WHO), the annual incidence of Mycobacterium Tuberculosis in 2018 was 10 million with an annual mortality of 1.5 million worldwide, which includes 251,000 deaths with HIV. HIV infection, IV drug abuse, and modern immunosuppression brought to attention multidrug-resistant (MDR) Mycobacteria tuberculosis. During the previous century, surgical techniques such as collapse therapy, intrapleural pneumothorax, plumbago, and thoracoplasty were employed and were mostly replaced by modern anti-tuberculous therapy. The development of MDR (multidrug resistance) tuberculosis and its complications, led to a small but renewed role for surgery in the treatment arsenal for complicated tuberculosis. Failure of conservative management with tube thoracostomy, prolonged aggressive anti-tuberculotic chemotherapy, any surgical drainage will lead to window thoracoplasty (Clagget, Eloesser) with subsequent thoracomyoplasty. Available muscle flaps are latissimus dorsi muscle, serratus anterior muscle, pectoralis major, and rectus abdominis muscle. Often, the omentum can be tunneled from the abdominal cavity trans-diaphragmatically to fill the necessary space. It is important to consider that these patients are usually malnourished and debilitated, and their nutritional status should be addressed appropriately often with feeding gastrostomy [28].

**Figure 10:**
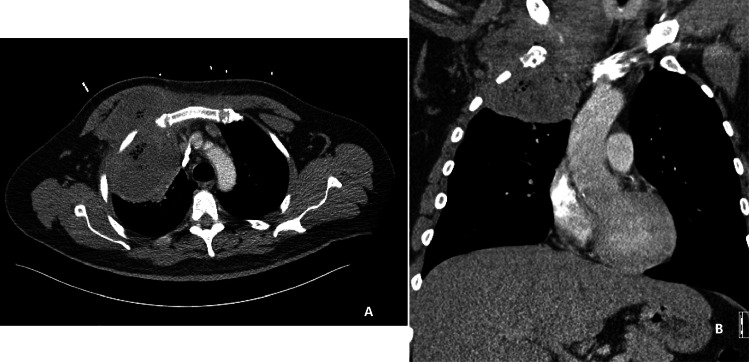
CT scan chest with empyema necessitans. (A) Axial, (B) Coronal: 48 yr M with uncontrolled DM showing large medium-density peripherally enhancing infected hematoma centered around a fractured right first rib wall with intrathoracic and chest wall components. Findings are most compatible large right-sided empyema secondary to infected intrapleural hematoma found to be tracking to skin with internal air representing necrosis consistent with empyema necessitans. The patient underwent chest wall exploration and pleural space drainage for optimization of drainage. Images Courtesy by Dr. Ory Wiesel

Empyema Necessitans: Empyema necessitans (EN) is a rare complication of pleural space infections and occurs when the infected fluid dissects spontaneously into the chest wall from the pleural space forming a fistula between the pleural cavity and skin (Figures 10A, 10B). EN results from the poorly or uncontrolled long-term complication of empyema and usually occurs after necrotizing pneumonia or pulmonary abscess. Pleural effusion with empyema necessitans is usually caused by Mycobacterium tuberculosis and Actinomyces israelii [29]. Other microbial causes include Pneumococci, Staph aureus, and Proteus. Early diagnosis and drainage of pleural effusion would prevent the development of empyema necessitans. The management consists of antimicrobials, tube drainage, incision, and drainage of the subcutaneous abscess together with drainage of the infected pleural space, and finally debridement, and decortication for obliterating the cavity to prevent fibrosis and facilitate lung expansion [29].

Summary: temporal stages and treatments modalities

**Figure 11:**
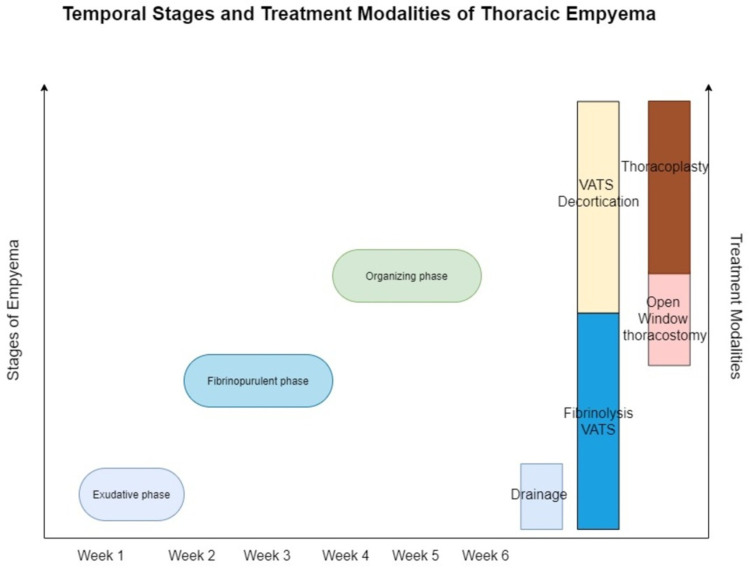
Time-based stages of empyema and the various treatment modalities for the management of various stages. VATS - Video-assisted thoracoscopic surgery

**Figure 12:**
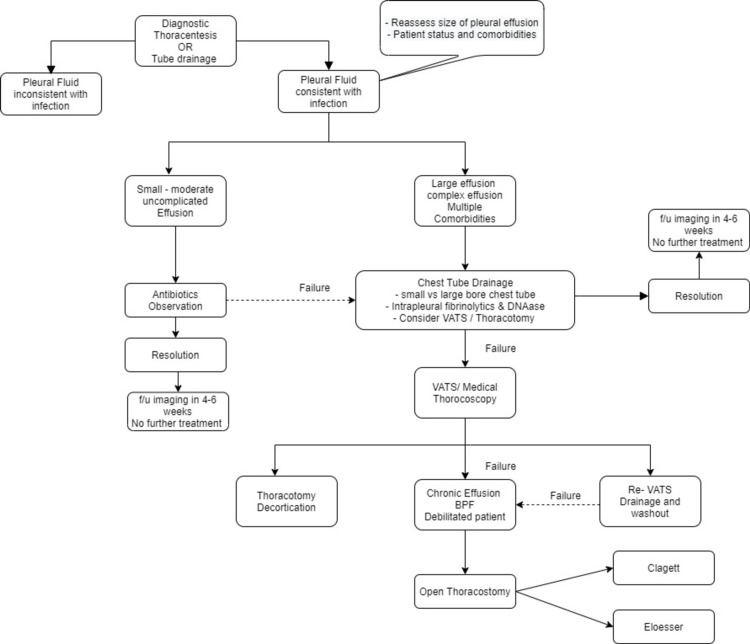
A schematic flow chart summarizing the various treatment modalities available for managing pleural infection and various stages where each of them may be used. Decisions regarding the timing of each treatment option may vary according to institutional expertise

The management principles for pleural space infections will require better patient risk categorization which will facilitate in adapting pertinent, prompt, pragmatic, evidence-based, stepwise, and multidisciplinary approaches. The various available treatment modalities if used timely at appropriate stages of parapneumonic effusions; will reduce the morbidity resulting from this disease (Figures 11, 12). 

Future directions

Empyema has traditionally been a surgical disease. Empiric antibiotics for all patients with pleural space infections and tube thoracotomy for select patients are the mainstay of initial management. According to ACCP 2000 guidelines; IPF, VATS, and thoracotomy with or without decortication are acceptable treatment modalities for suitable candidates. BTS 2010 recommends early consultation with a thoracic surgeon for any possible surgical options for persistence or worsening of parapneumonic effusions and empyema. AATS 2017 recommends considering VATS as the first-line approach for stage 2 acute empyema. In spite of the increasing use of IPF with no systemic hemorrhagic risks, routine use of IPF is not recommended according to BTS and AATS guidelines [3,16,17]. There has been researching with lesser tPA dose to minimize the expense and yet maintain similar safety and efficacy profile. Additionally, the use of combined tPA and DNase over 6 doses in frail and poor surgical candidates has been investigated as a salvage treatment modality. Large RCTs in these areas are required to validate the use of IPF for a suitable patient population. One such ongoing RCT is MIST3 trial which may bring a paradigm shift in the management of parapneumonic effusion by demonstrating whether the early IPF regimen is similar or better than early VATS in appropriately select patient population. This will also mean empyema; a historically surgical disease will have medical modalities as the first-line therapy. Open thoracotomy has been reserved for patients who failed other modalities and sometimes delays to surgery increase the risks of conversion from VATS to open thoracotomy. However, to this day there have not been any RCT studies providing data to assess criteria for patient selection, the timing of surgical intervention, or whether patient outcomes are truly enhanced by surgery. There have been recent advances in establishing validated scoring systems as predictive models to stratify patients into risk categories. One such model is the RAPID score system developed by Rahman et al. to assess three-month mortality and hospital length of stay for patients with pleural effusions using the MIST1 and two cohorts [19]. However, there is a need to extend such a scoring system to assist clinicians in decision-making regarding choosing a specific modality of treatment for specific risk categories. More studies to apply this score to the outpatient settings and assess the need for de-escalation and tapering of antibiotic course is underway (NCT04615286) [19]. The use of medical thoracoscopy as a therapeutic modality to allow lysis of adhesions and facilitate chest tube drainage of pleural fluid has been studied in smaller patient series showing success rates and not requiring any further interventions. Combining medical thoracoscopy with IPF has also been undertaken in small retrospective studies. However, it has not been incorporated into any recommended guidelines yet. A multicenter RCT, SPIRIT trial, designed to study the feasibility of medical thoracoscopy as an early treatment strategy for pleural infection is ongoing and might shed light on less invasive interventional options. PACT study has been designed to introduce ambulatory management strategies for pleural infections. One of the workstreams is focused on studying the effectiveness of antibiotics’ ability to penetrate pleural space which will facilitate de-escalation of antibiotics, hence assisting in antibiotic stewardship and reducing the emergence of resistant organisms [30].

## Conclusions

Early diagnosis and initiating appropriate treatment are crucial to prevent further complications from parapneumonic effusions. Most of the early treatment strategies that stemmed from expert opinions have become the current day evidence-based management guidelines. While conservative approaches with antibiotics and tube drainage are empirical treatments for pleural effusions, the treatment approach for more complicated parapneumonic effusions is IPF, VATS, and open thoracotomy in the select patient population. Recently, there are several national, multicenter studies and RCTs being conducted to expand the risk stratification models and to support a choice of appropriate modalities for suitable patients in a timely manner. Continued advances in the use of IPF as salvage treatment for frail and poor surgical candidates, IPF along with medical thoracoscopy, therapeutic thoracocentesis for suitable candidates, and a better understanding of antibiotics’ penetration into pleural space might help modify the current standards of care. The authors are of the opinion that in the future there will be a shift in empyema treatment guidelines from surgical management to a medicine-first treatment modality; hence changing the clinician's outlook about empyema management overall.
